# Sleep Disorder Symptoms Among Adults in 8 States and the District of Columbia, 2017

**DOI:** 10.5888/pcd18.210305

**Published:** 2021-12-30

**Authors:** Yong Liu, Susan A. Carlson, Anne G. Wheaton, Kurt J. Greenlund, Janet B. Croft

**Affiliations:** 1 Division of Population Health, Centers for Disease Control and Prevention, Atlanta, Georgia

## Abstract

Sleep disorder symptoms (trouble falling asleep or staying asleep, unintentionally falling asleep, snoring loudly, and episodes of having stopped breathing) among US adults (N = 59,108) from 8 states and the District of Columbia were analyzed by using data from the 2017 Behavioral Risk Factor Surveillance System. We conducted a multivariable logistic regression to assess the association between the 4 symptoms and sociodemographic characteristics, risk behaviors, and chronic conditions. The 4 symptoms were prevalent and more likely to be reported among adults with any chronic condition(s) than their counterparts without symptoms and among those who slept fewer than 7 hours compared with those who slept 7 to 9 hours.

**Summary**

**What is already known on this topic?**

Sleep disorders are associated with chronic conditions. Limited population-level data are available on sleep disorder symptoms.

**What is added in this report?**

Among adults in 8 states and the District of Columbia in 2017, 49.5% reported having trouble falling or staying asleep, 23.1% reported having unintentionally fallen asleep, 41.1% reported snoring loudly, and 13.6% reported having stopped breathing. All sleep disorder symptoms were more prevalent among adults who had a chronic condition or short sleep duration.

**What are the implications for public health practice?**

Enhancing surveillance of sleep disorder symptoms among US adults can help promote awareness among public health practitioners and health care professionals to improve sleep health.

## Objective

Sleep disorders are associated with low sociodemographic characteristics, health-risk behaviors, and chronic conditions (1). However, limited population-level information exists on sleep disorder symptoms. Our study aimed to present estimates of sleep disorder symptoms including sleep satisfaction or quality, alertness, snoring, and observed apnea among adults (2).

## Methods

The Behavioral Risk Factor Surveillance System (BRFSS) is an annual, state-based, random-digit–dialed telephone interview survey designed to monitor health conditions and risk behaviors in a representative sample of adults (aged ≥18 years) in all 50 states, the District of Columbia, and US territories. In 2017, 8 states (Arizona, Kansas, Minnesota, Nebraska, Nevada, North Dakota, Oregon, Tennessee) and the District of Columbia administered an optional module about sleep disorder symptoms in addition to a core module including sociodemographic and health characteristics ( www.cdc.gov/brfss). We considered sleep disorder symptoms as experiencing 1 or more days in the past 14 days of 1) trouble falling asleep, staying asleep, or sleeping too much, or 2) unintentionally falling asleep. Sleep disordered breathing symptoms were defined as 3) you have been told that you snored loudly, or 4) someone observed that you stopped breathing during sleep. Age-adjusted prevalence of each symptom was standardized to the year 2000 projected US adult population (3). Adjusted prevalence ratios and 95% CIs were estimated by using multivariable logistic regression models controlling for sex, age group, race and ethnicity, marital status, body mass index (BMI) category, any chronic disease (including heart disease [coronary heart disease or angina, or myocardial infarction or heart attack], stroke, diabetes, arthritis, history of asthma, chronic obstructive pulmonary disease, cancer [not including skin cancer], and chronic kidney disease), high blood pressure, depression, and average sleep duration (<7 h, 7–9 h, and >9 h sleep in a 24-h period). High blood pressure and depression, which have characteristics different from other chronic diseases, were included separately in the study, although all chronic conditions are strongly associated with sleep disorders (1,4,5). Among the 76,542 adult respondents, 59,108 with complete information were included in this study. Only significant differences are presented (*P < .05*)*.*

## Results

The age-adjusted prevalence was 49.5% for trouble falling or staying asleep, 23.1% for unintentionally falling asleep, 41.1% for snoring loudly, and 13.6% for having stopped breathing (Table 1 and Table 2). Three in 4 adults (74.9%) reported having any sleep disorder symptom; 38.2% reported 1, 24.6% reported 2, 9.8% reported 3, and 2.3% reported all 4 symptoms. Men were less likely to report trouble falling asleep or staying asleep but were more likely to report snoring or having stopped breathing than women. Adults aged 45 to 64 and 65 or older were less likely to report trouble falling asleep or staying asleep but more likely to report snoring or having stopped breathing than adults aged 18 to 44 years. Adults aged 65 years or older were more likely to report unintentionally falling asleep than those aged 18 to 44. Non-Hispanic Black and Hispanic adults were less likely to report trouble falling asleep or staying asleep but more likely to report unintentionally falling asleep than non-Hispanic White adults. Divorced, widowed, separated, or never married adults were more likely to report trouble falling asleep or staying asleep or unintentionally falling asleep but less likely to report snoring than married adults or members of an unmarried couple. Breathing symptom prevalence increased with increasing BMI. In addition, a higher prevalence was shown for each symptom among adults with any chronic disease, high blood pressure, or depression than their counterparts without symptoms, or who slept less than 7 hours compared with those who slept 7 to 9 hours. A U-shaped relationship between sleep duration and unintentionally falling asleep was observed. All significant differences remained after controlling for covariates.

**Table 1:** Age-Adjusted Prevalence^a^ and Adjusted Prevalence Ratio^b^ of Sleep Disorder Symptoms Among Adults in 8 States^c^ and the District of Columbia, 2017 Behavioral Risk Factor Surveillance System

Characteristic	Number of respondents	Trouble falling asleep or staying asleep^d^		Unintentionally falling asleep^e^	
	Unweighted, n	Prevalence (95% CI)^a^	Prevalence ratio (95% CI)^b^	Prevalence (95% CI)^a^	Prevalence ratio (95% CI)^b^
**Crude percentage**	59,108	49.1 (48.4–49.8)	NA	24.0 (23.4–24.5)	NA
**Age-adjusted percentage**	59,108	49.5 (48.8–50.2)		23.1 (22.5–23.7)	
**Sex**					
Male	27,657	43.8 (42.8–44.8)	0.80 (0.78–0.83)	23.0 (22.2–23.8)	1.05 (0.99–1.10)
Female	31,451	55.3 (54.3–56.3)	1.0 [Reference]	23.4 (22.5–24.2)	1.0 [Reference]
**Age group, y**					
18–44	16,150	52.0 (50.8–53.1)	1.0 [Reference]	21.2 (20.2–22.1)	1.0 [Reference]
45–64	21,861	50.4 (49.3–51.4)	0.94 (0.91–0.98)	21.3 (20.4–22.2)	0.98 (0.92–1.05)
≥65	21,097	41.6 (40.5–42.6)	0.76 (0.73–0.80)	33.4 (32.4–34.4)	1.60 (1.49–1.72)
**Race and ethnicity**					
Non-Hispanic White	48,909	51.2 (50.4–52.0)	1.0 [Reference]	21.1 (20.5–21.7)	1.0 [Reference]
Non-Hispanic Black	3,426	44.6 (41.3–47.8)	0.78 (0.72–0.85)	32.8 (29.9–35.9)	1.40 (1.26–1.56)
Hispanic	3,612	41.9 (39.8–44.1)	0.86 (0.81–0.91)	23.8 (22.0–25.6)	1.20 (1.11–1.31)
Other non-Hispanic^f^	3,161	49.6 (46.7–52.4)	0.93 (0.87–1.00)	28.7 (26.2–31.4)	1.36 (1.23–1.51)
**Marital status**					
Married or member of an unmarried couple	33,627	47.0 (45.9–48.1)	1.0 [Reference]	20.3 (19.4–21.2)	1.0 [Reference]
Divorced, widowed, or separated	15,889	52.1 (48.8–55.4)	1.04 (1.01–1.08)	26.6 (24.1–29.2)	1.19 (1.12–1.26)
Never married	9,592	53.8 (52.0–55.6)	1.21 (1.17–1.26)	27.0 (25.4–28.7)	1.43 (1.33–1.53)
**Body mass index (kg/m^2^)**					
Underweight or normal weight (<25.0)	19,034	49.7 (48.5–50.9)	1.0 [Reference]	19.8 (18.9–20.8)	1.0 [Reference]
Overweight (25.0–29.9)	21,549	47.0 (45.8–48.3)	0.97 (0.93–1.00)	21.3 (20.4–22.4)	1.02 (0.96–1.09)
Obese (≥30.0)	18,525	52.5 (51.2–53.8)	0.97 (0.94–1.01)	28.4 (27.2–29.5)	1.23 (1.15–1.31)
**Selected chronic disease** g					
Yes	31,010	58.3 (57.0–59.5)	1.19 (1.15–1.23)	29.0 (27.9–30.2)	1.40 (1.33–1.48)
No	28,098	43.3 (42.4–44.1)	1.0 [Reference]	18.2 (17.5–18.9)	1.0 [Reference]
**High blood pressure**					
Yes	22,946	57.2 (55.3–59.0)	1.09 (1.06–1.13)	29.2 (27.4–31.0)	1.16 (1.09–1.22)
No	36,037	47.2 (46.4–48.0)	1.0 [Reference]	20.6 (19.9–21.2)	1.0 [Reference]
**Depression**					
Yes	11,704	72.2 (70.9–73.5)	1.54 (1.50–1.58)	31.5 (30.2–32.9)	1.35 (1.28–1.43)
No	47,187	43.3 (42.5–44.1)	1.0 [Reference]	20.8 (20.1–21.4)	1.0 [Reference]
**Average sleep duration in a 24-hour period, h**					
<7	18,054	62.1 (60.9–63.3)	1.44 (1.40–1.48)	29.1 (28.0–30.2)	1.39 (1.32–1.46)
7–9	39,012	42.7 (41.8–43.6)	1.0 [Reference]	19.3 (18.6–20.0)	1.0 [Reference]
>9	2,042	50.1 (45.4–54.8)	1.01 (0.92–1.11)	32.1 (28.0–36.5)	1.32 (1.15–1.50)

Abbreviation: NA, not applicable.

a Age-adjusted prevalence and 95% CIs were standardized to the 2000 projected US population for all selected characteristics except for age group.

b Adjusted prevalence ratio was obtained from multivariable logistic regression models with all covariates in the table.

c Eight states are Arizona, Kansas, Minnesota, Nebraska, Nevada, North Dakota, Oregon, and Tennessee.

d Trouble falling asleep or staying asleep was defined as a respondent reporting ≥1 day of trouble falling asleep or staying asleep or sleeping too much in the past 14 days.

e Unintentionally falling asleep was defined as a respondent reporting ≥1 day of unintentionally falling asleep in the past 14 days.

f Other, non-Hispanic includes non-Hispanic American Indian or Alaskan Native, non-Hispanic Asian, non-Hispanic Native Hawaiian or Pacific Islander, non-Hispanic other race, and non-Hispanic multiracial.

g Chronic diseases include heart disease (coronary heart disease/angina, myocardial infarction/heart attack), stroke, diabetes, arthritis, history of asthma, chronic obstructive pulmonary disease, cancer (not including skin cancer), and chronic kidney disease.

**Table 2:** Age-Adjusted Prevalence^a^ and Adjusted Prevalence Ratio^b^ of Sleep Disordered Breathing Symptoms Among Adults in 8 States^c^ and the District of Columbia, 2017 Behavioral Risk Factor Surveillance System

Characteristic	Number of respondents	Snoring loudly^d^		Observed breathing stopped^e^	
	Unweighted, n	Prevalence (95% CI)^a^	Prevalence ratio (95% CI)^b^	Prevalence (95% CI)^a^	Prevalence ratio (95% CI)^b^
Crude percentage	59,108	41.8 (41.1–42.4)	NA	14.2 (13.7–14.6)	NA
Age-adjusted percentage	59,108	41.1 (40.4–41.7)		13.6 (13.1–14.0)	
**Sex**					
Male	27,657	50.3 (49.3–51.3)	1.61 (1.56–1.67)	17.8 (17.1–18.5)	2.23 (2.07–2.41)
Female	31,451	31.6 (30.7–32.5)	1.0 [Reference]	9.4 (8.9–10.0)	1.0 [Reference]
**Age group, y**					
18–44	16,150	34.4 (33.3–35.5)	1.0 [Reference]	9.5 (8.9–10.2)	1.0 [Reference]
45–64	21,861	50.6 (49.6–51.7)	1.27 (1.22–1.33)	18.2 (17.4–19.0)	1.34 (1.21–1.48)
≥65	21,097	43.0 (42.0–44.1)	1.07 (1.02–1.13)	17.1 (16.3–17.9)	1.22 (1.09–1.37)
**Race and ethnicity**					
Non-Hispanic White	48,909	40.7 (40.0–41.5)	1.0 [Reference]	13.7 (13.2–14.3)	1.0 [Reference]
Non-Hispanic Black	3,426	42.5 (39.3–45.7)	1.06 (0.97–1.15)	13.3 (11.4–15.5)	0.90 (0.75–1.08)
Hispanic	3,612	42.5 (40.3–44.6)	1.01 (0.95–1.07)	11.8 (10.4–13.3)	0.84 (0.74–0.97)
Other, non-Hispanic^f^	3,161	39.8 (37.0–42.6)	0.99 (0.91–1.07)	14.6 (12.7–16.8)	1.06 (0.90–1.25)
**Marital status**					
Married or member of an unmarried couple	33,627	43.5 (42.4–44.5)	1.0 [Reference]	13.6 (13.0–14.2)	1.0 [Reference]
Divorced, widowed,or separated	15,889	38.3 (35.7–41.0)	0.85 (0.81–0.88)	15.8 (14.3–17.5)	1.01 (0.93–1.10)
Never married	9,592	37.2 (35.4–39.0)	0.72 (0.67–0.76)	12.5 (11.3–13.8)	0.72 (0.64–0.81)
**Body mass index (kg/m^2^)**					
Underweight or normal weight (<25.0)	19,034	26.4 (25.3–27.4)	1.0 [Reference]	6.7 (6.1–7.3)	1.0 [Reference]
Overweight (25.0–29.9)	21,549	41.4 (40.3–42.6)	1.42 (1.36–1.49)	10.7 (10.1–11.4)	1.39 (1.25–1.55)
Obese (≥30.0)	18,525	55.9 (54.6–57.1)	1.96 (1.87–2.05)	23.3 (22.3–24.3)	2.92 (2.63–3.23)
**Selected chronic disease** g					
Yes	31,010	46.0 (44.7–47.2)	1.12 (1.08–1.16)	18.9 (18.0–19.9)	1.61 (1.49–1.75)
No	28,098	37.3 (36.5–38.2)	1.0 [Reference]	9.1 (8.6–9.7)	1.0 [Reference]
**High blood pressure**					
Yes	22,946	51.9 (50.2–53.7)	1.19 (1.15–1.24)	22.3 (20.9–23.7)	1.55 (1.43–1.67)
No	36,037	36.7 (36.0–37.5)	1.0 [Reference]	9.9 (9.4–10.3)	1.0 [Reference]
**Depression**					
Yes	11,704	46.8 (45.3–48.2)	1.22 (1.17–1.27)	21.9 (20.7–23.1)	1.87 (1.73–2.02)
No	47,187	39.5 (38.7–40.3)	1.0 [Reference]	11.3 (10.8–11.8)	1.0 [Reference]
**Average sleep duration in a 24-hour period, h**					
<7	18,054	44.4 (43.2–45.6)	1.09 (1.05–1.13)	16.9 (16.1–17.8)	1.32 (1.22–1.42)
7–9	39,012	38.9 (38.1–39.8)	1.0 [Reference]	11.5 (10.9–12.0)	1.0 [Reference]
>9	2,042	42.2 (38.1–46.5)	1.03 (0.93–1.14)	16.2 (13.5–19.4)	1.11 (0.90–1.37)

Abbreviation: NA, not applicable.

a Age-adjusted prevalence and 95% CIs were standardized to the 2000 projected US population for all selected characteristics except for age group.

b Adjusted prevalence ratio was obtained from multivariable logistic regression models with all covariates in the table.

c Eight states are Arizona, Kansas, Minnesota, Nebraska, Nevada, North Dakota, Oregon, and Tennessee.

d Snoring loudly was defined as an affirmative response to “Have you ever been told that you snore loudly?”

e Observed breathing stopped was defined as an affirmative response to “Has anyone ever observed that you stop breathing during your sleep?”

f Other, non-Hispanic includes non-Hispanic American Indian or Alaskan Native, non-Hispanic Asian, non-Hispanic Native Hawaiian or Pacific Islander, non-Hispanic other race, and non-Hispanic multiracial.

g Chronic diseases include heart disease (coronary heart disease/angina, myocardial infarction/heart attack), stroke, diabetes, arthritis, history of asthma, chronic obstructive pulmonary disease, cancer (not including skin cancer), and chronic kidney disease.

## Discussion

Sleep disorder symptoms were common among adults in the 8 states and the District of Columbia in 2017. About 4 in 10 adults reported snoring, consistent with a previous study from 12 states in the 2009 BRFSS (48.0%) and the 2005–2006 National Health and Nutrition Examination Survey (NHANES) (47.9%) (6,7); however, prevalence for unintentionally falling asleep was lower than in an earlier BRFSS study (23.1% vs 37.9%), likely because of a shorter recall period used (2 weeks versus 30 days) (6). In addition, differences in prevalence by sociodemographic and health characteristics were observed for all 4 sleep disorder symptoms. For example, older adults were more likely to report 3 of the 4 symptoms. Our observation of a positive relationship between all 4 sleep disorders and any selected chronic disease, high blood pressure, depression, or short sleep duration was like that of previous studies (4,5,8,9).

Evidence-based interventions can improve sleep health, especially among groups at high risk for disease, such as older adults or those with short sleep duration, those who are overweight or obese, and those with any chronic condition. Educating primary care professionals or physicians to address sleep disorder symptoms for adults with obesity or chronic conditions could improve patients’ sleep health (10). Comprehensive public health strategies, such as enhancing surveillance and research and promoting public awareness about sleep disorders, are needed to improve sleep health at the population level. Snoring and apnea were more likely to be reported by adults who were married or a member of an unmarried couple, highlighting that sleep partners whose sleep might also be negatively affected can play a role in the diagnosis and management of sleep (11). Therefore, it is critical that adults recognize these symptoms and discuss them with their primary care provider who can then make referrals to a sleep specialist.

This study has several limitations. First, sleep disorder symptoms were based on self-report and were not verified with objective sleep studies. Furthermore, the 2 breathing symptom questions relied on observation by another individual, which could partially explain a higher prevalence of the symptoms among married adults than among never-married ones. Second, sleep disorder symptoms reflect retrospective conditions and might have recall bias, although the current study used a shorter recall period than previous studies, which might have resulted in greater accuracy (12). Third, our results only represent noninstitutionalized adults in 8 states and the District of Columbia. Finally, nonresponse bias, because of low response rates, may have influenced our results.

Sleep health should be assessed at the population level by using measures of the 4 sleep disorder symptoms to help monitor and promote sleep health in our nation.
